# Spin polarization in the phase diagram of a Li–Fe–S system

**DOI:** 10.1038/s41598-019-56244-x

**Published:** 2019-12-27

**Authors:** Tsuyoshi Takami, Tomonari Takeuchi, Toshiharu Fukunaga

**Affiliations:** 1 Office of Society-Academia Collaboration for Innovation, Center for Advanced Science Innovation, Kyoto University, Gokasho, Uji, Kyoto, 611-0011 Japan; 2 Research Institute of Electrochemical Energy (RIECEN), National Institute of Advanced Industrial Science and Technology (AIST), 1-8-31 Midorigaoka, Ikeda, Osaka, 563-8577 Japan

**Keywords:** Chemistry, Materials for energy and catalysis

## Abstract

Divalent and trivalent states of Fe ions are known to be stable in inorganic compounds. We focus a novel Li_*x*_FeS_5_ cathode, in which the Li content (*x*) changes from 2 to 10 by an electrochemical technique. As *x* increases from 2, a Pauli paramagnetic conductive Li_2_FeS_5_ phase changes into a superparamagnetic insulating Li_10_FeS_5_ phase. Density functional theory calculations suggest that Fe^+^ ions in a high-*x* phase are responsible for ferromagnetic spin polarization. Reaching the monovalent Fe ion is significant for understanding microscopic chemistry behind operation as Li-ion batteries and the original physical properties resulting from the unique local structure.

## Introduction

Since iron is one of the most ubiquitous and stable elements on our planet, various Fe compounds are used for many fields such as a metal, alloys and organic/inorganic compounds. Fe ions are in variety valence states from 0 to +6 in organic compounds^1–4^. However, the valence state of Fe ions is generally divalent or trivalent in inorganic compounds. As unconventional high-valence examples, SrFe^4+^O_3_ and CaFe^4+^O_3_ were synthesized under high pressures^5,6^. Furthermore, in complex oxides, charge transfer leads to Fe^3.75+^ in LaCu_3_Fe_4_O_12_ ^7^. On the contrary, an unusual low-valence state beyond the constraint is expected to be formed by cation injection in an inorganic host lattice, as in the case for anion injection.

**Figure 1:**
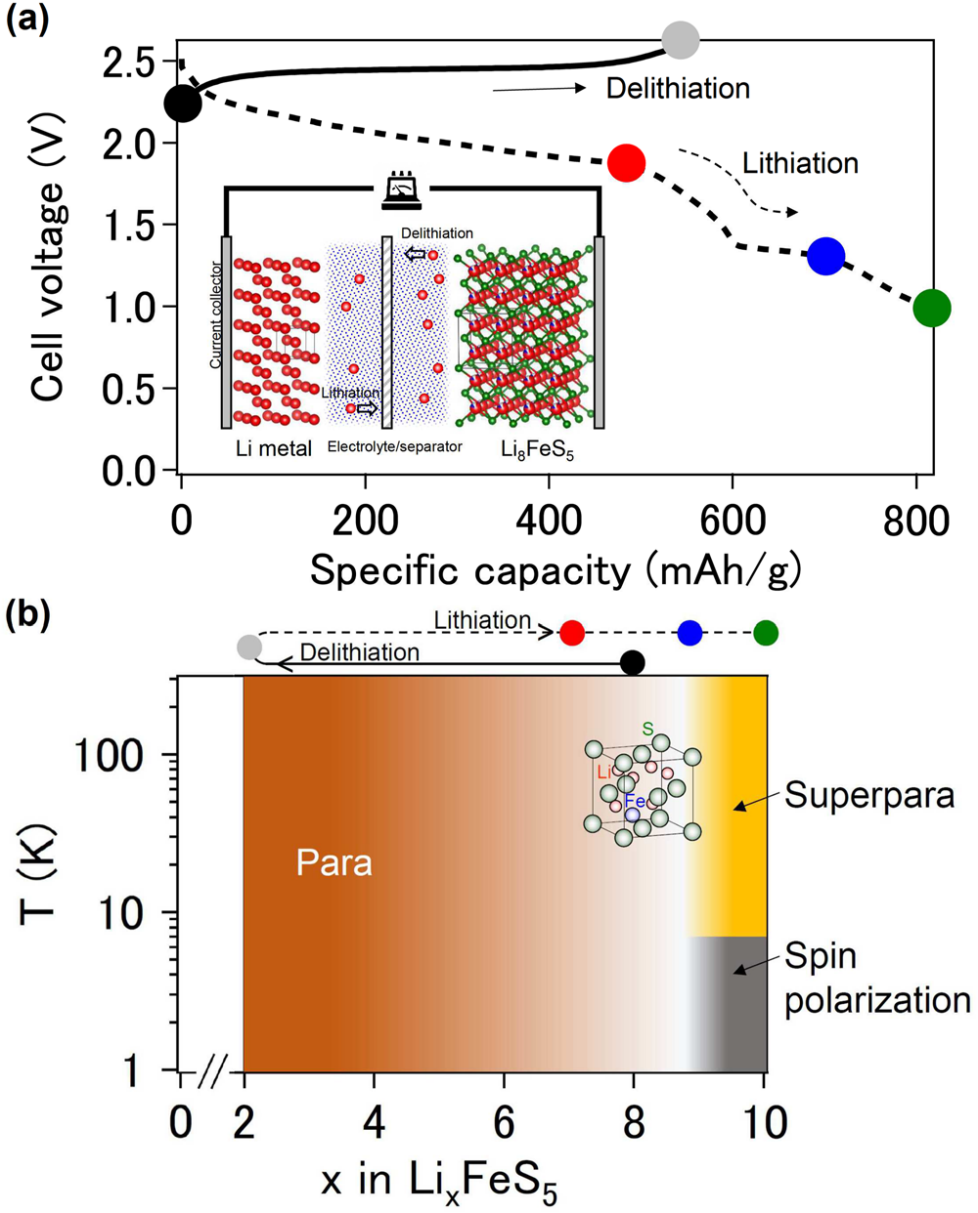
(**a**) Delithiation (solid) and lithiation (dashed) profiles for the Li_8_FeS_5_/C electrode at 298 K. The lithiation plateaus at approximately 2.0 V and 1.4 V correspond to the reduction of sulfur and Fe–S components, respectively. Inset: Schematic illustration of the Li_8_FeS_5_/Li cell. Li, Fe and S atoms are represented as red, blue and green spheres, respectively. (**b**) Schematic phase diagram of the Li–Fe–S system. The color symbols correspond to those in (**a**).

The recent discovery of the Li_8_FeS_5_ system with an exceptionally high capacity of 800 mAh/g^8^, which is a fingerprint for up to eight transferrable Li ions (Fig. 1a), gives opportunities to achieve an unusual valence state of the Fe ions owing to a wide range of Li contents (*x* = 2 –10 in Li_*x*_FeS_5_). For example, this means that the valence state of the Fe ions naturally decreases with increasing *x* in order to keep charge neutrality. The number of transferrable Li ions per unit cell is 6.4, which is the largest value to the best of our knowledge. Also, unique amorphous (*x* = 2)/low-crystalline (*x* = 10) transformations are induced by the transferrable Li ions^8^. The precise structure of the amorphous phase has remained unknown, and X-ray diffraction (XRD), extended X-ray absorption fine structure (EXAFS) and X-ray absorption near-edge structure (XANES) measurements indicated the formation of Fe–S bond and the presence of discrete sulfer ions^8^. Besides such *x* variation and structural change in Li_*x*_FeS_5_, the local structure, the magnetic properties and the electronic properties are expected to be drastically altered by *x*, while there are no available data on them.

We therefore select the system as an underexplored but intriguing target, and report the magnetic and electronic properties of the products after Li ion propagation out of (delithiation) and into (lithiation) the pristine material by bulk magnetization, scanning spreading resistance microscopy (SSRM), ultraviolet photoemission spectroscopy (UPS) and magnetic force microscopy (MFM) measurements. We prepared, in a first step, several materials by fixing *x* in Li_*x*_FeS_5_ using an electrochemical technique and investigated their magnetic properties to obtain an overview of the phase diagram. Perturbations by Li ions that have no net magnetization of their own transformed the pristine phase into a Pauli paramagnetic (PM) phase and a superparamagnetic (SPM) phase (Fig. 1b). In a second step, the two end members (*x* = 2 and 10) were studied in detail to understand their electronic properties, electronic states and local structures. As a result, the Li_*x*_FeS_5_ system was found to exhibit electronic phase switching between a low-*x* conductive state and a high-*x* insulating state. Counter to our common belief, density functional theory (DFT) calculations suggest the monovalent Fe ion for *x* = 9.1 plays key roles in spin polarization.

## Results and Discussion

### Magnetic properties

The magnetization data were collected at five stages in the voltage profile (Fig. 1a). The *χ*(*T*) curve of pristine Li_8_FeS_5_ followed the Curie – Weiss law and yielded an effective magnetic moment (*μ*_eff_) of 3.7 *μ*_B_, where *μ*_B_ is the Bohr magneton. Both XRD analyses and high-energy X-ray total scattering measurements indicated that the pristine material was Fe-substituted (Li, Fe)_2_S^9^. The estimated *μ*_eff_ was consistent with *μ*_eff_ = 3.9 *μ*_B_ of Li_8_FeS_5_ (=(Li_0.8_Fe_0.1_)_2_S) with Li off-stoichiometry, in which the Fe^2+^ ions in the FeS_4_ tetrahedra are in a mixed state between an intermediate-spin state and a high-spin state.

**Figure 2:**
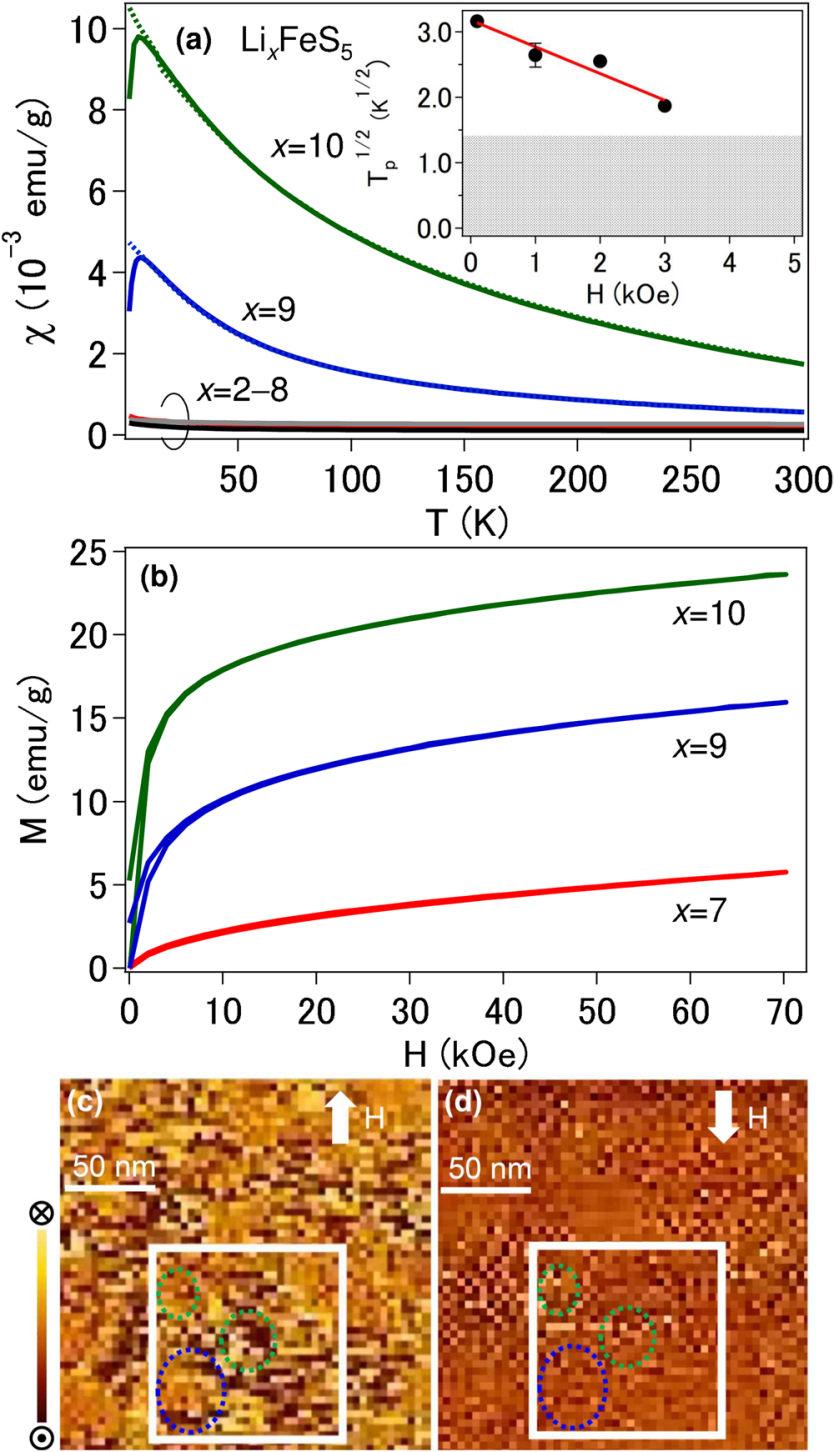
(**a**) Temperature dependence of magnetic susceptibility measured in the ZFC mode (solid) and the FC mode (dotted) in a field of 1 kOe. The color corresponds to that in Fig. 1a. Inset: Magnetic field dependence of *T*_*p*_ for *x* = 10. The solid line is the result of fitting *T*_*p*_ ∝ [1 – (*H*/*H*_0_)]^2^^10^ to the data. Above 4 kOe, *T*_*p*_ was not observed down to 2 K. The temperature range below 2 K is described by gray. (**b**) Plot of 2 K magnetization versus magnetic field. (**c,d**) Two 200 nm × 200 nm MFM images taken of the same area at 150 K (>*T*_*p*_) after lithiation (*x* = 10). A magnetic field of 5 kOe is applied (**c**) along the arrow and (**d**) in the reversed direction. Scale bar: 50 nm; MFM z-range: 3.6°. The frizzy or fuzzy appearance induced by the scanning process is visible, but we cannot exclude that part. The squares show the MFM images over a 100 nm × 100 nm area.

Upon delithiation process, the Li ions were extracted from the Li_8_FeS_5_ host lattice (inset of Fig. 1a). The *χ*(*T*) curve for Li_2_FeS_5_ showed a Pauli-like lack of temperature dependence down to 100 K. The density of states at the Fermi level, *N*(*E*_F_), was estimated as 6.8 × 10^23^ eV^−1^ cm^−3^ using the relation *χ*_0_ = *μ*_B_^2^*N*(*E*_F_), where *χ*_0_ is the temperature independent *χ*. On the contrary, the *χ* for *x* ≥ 9 in Li_*x*_FeS_5_ increased during lithiation (Fig. 2a) and there was a branch between the zero-field cooling (ZFC) and field cooling (FC) data at low temperature (*T*_irr_). A cusp at a lower temperature in the ZFC curve appeared after the second plateau at approximately 1.4 V during the lithiation process (Fig. 1a). The peak temperature (*T*_p_) for Li_10_FeS_5_ was 10 K at 100 Oe and shifted to lower temperatures with increasing magnetic field, maintaining the relationship *T*_p_ < *T*_irr_. Such field variation of T_*p*_ is described by the power law, T_*p*_ ∝ [1 − (*H*/*H*_0_)]^2^^10^ characteristic of superparamagnetism rather than antiferromagnetism (inset of Fig. 2a). Here, superparamagnetism appears in ferromagnetic (FM) or ferrimagnetic nanoscale particles. Magnetization analyses above 250 K where the Curie-like contribution with a Curie temperature of 90 K is dominant indicate the formation of magnetic polarons with an unusually high spin quantum number (S = 16), assuming that the g factor is 2 (Fig. S1). The concave, rather than convex, shape of the *χ*(*T*) curve also supports this scenario^11^.

The magnetization at 2 K after the second plateau approached saturation at higher magnetic fields did not completely saturate even at 70 kOe (Fig. 2b). A magnetic hysteresis with a residual magnetization was observed and disappeared at 150 K (Fig. S2). As the particle size decreases to the nanoscale, the FM domain wall can no longer be defined. Thus, once an external field is removed above T_*p*_, the thermal fluctuation cancels the residual magnetization. Based on the *χ*(*T*) and *M*(*H*) curves, T_*p*_ was assigned as the blocking temperature characteristic of SPM behavior, where the thermal energy becomes comparable with the energy required for spins to freeze. Here, binding and conductive additives are extrinsic since they are nonmagnetic.

### MFM measurements

We obtained the FM spin polarization signal in the nanoparticles (NPs) after lithiation from the bulk magnetization measurements, but there was no evidence that the magnetic signal was intrinsic as this macroscopic technique once in a while captures signals originating from trivial sources. MFM is probably the most suitable method for investigating the SPM state as with the presence of NPs though we have used muons and neutrons to detect short/long-range FM order^12,13^. Dark and bright contrasts associated with the positive and negative phase shifts, respectively, were observed together with a high population of NPs (Fig. 2c,d). This indicates that the in-plane dipole moments arose from the NPs in the in-plane magnetic field. When the direction of the magnetic field is reversed, the contrast in the same region is reversed for the ideally isolated SPM NPs due to the flexible rotation of spins. In fact, the dark contrast turned the bright one with a magnetic field reversal and vice versa (green circles in Fig. 2c,d). The reason why the partial rotation is observed elsewhere is unclear, but the magnetic polarons quench the flexible rotation of spins. In particular, when one localized impurity spin and the carrier spins around the impurity form the magnetic polaron, the antiferromagnetic interaction between the localized impurities and the FM interaction between the magnetic polarons result in the small phase-shift change of the dark region, while remaining as the bright contrast (blue circles in Fig. 2c,d). There was also an area where the MFM image did not show a phase shift corresponding to the nonmagnetic acetylene black and binder. Since MFM measurements monitor a superposition of the magnetic and electrostatic signals, the displayed MFM data call into question the magnetic mechanism to some extent, but provide a plausible explanation reflecting most of the features of the FM spin-polarized NPs.

### Resistance measurements

**Figure 3:**
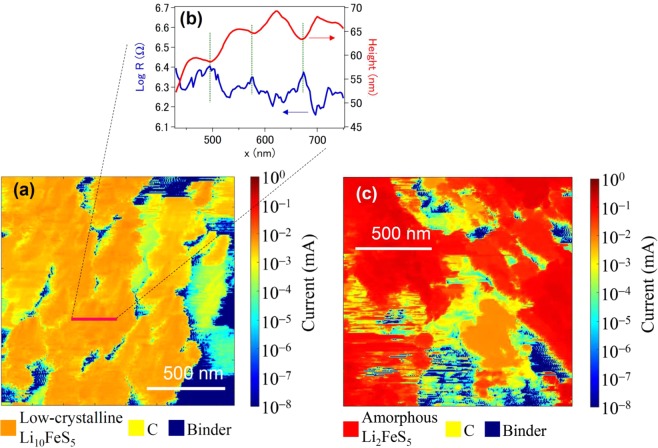
SSRM images of the samples after (**a**) lithiation and (**c**) delithiation. (**b**) Height and resistance profiles along the marked path. Scale bar: 500 nm.

After lithiation, the local electronic resistance was position-dependent and its span varied by six orders of magnitude even in the area with no height corrugations (Fig. 3a color bar). There were some regions with high (blue), medium (yellow) and low (orange) resistance originating from the binder, acetylene black and the active material (Li_10_FeS_5_), respectively. The light-blue region may also correspond to a phase produced by the decomposition of electrolyte solution. The local resistance of the active material was generally in the range of semiconductors, which rules out the possibility of metallic Fe being included. The height profiles showed a periodic array of NPs 5–10 nm in height (Fig. 3b). The electronic resistance exhibited a cusp at these interfaces (Fig. 3b). These results are consistent with the properties of NPs. Attempts to measure the Hall effect for the compressed pellet have failed because of a high resistivity.

In contrast, after delithiation, also shown in Fig. 3c are some regions with high (blue), medium (yellow) and low (red) resistance, which correspond to the binder, acetylene black and the active material (Li_2_FeS_5_), respectively. The region with lower resistances corresponded well with the distribution of Fe and S atoms (Fig. S3). The resistance of the active material after delithiation was almost one-fiftieth of that after lithiation. Most interestingly, the electrons were predominately delocalized.

### UPS measurements

**Figure 4:**
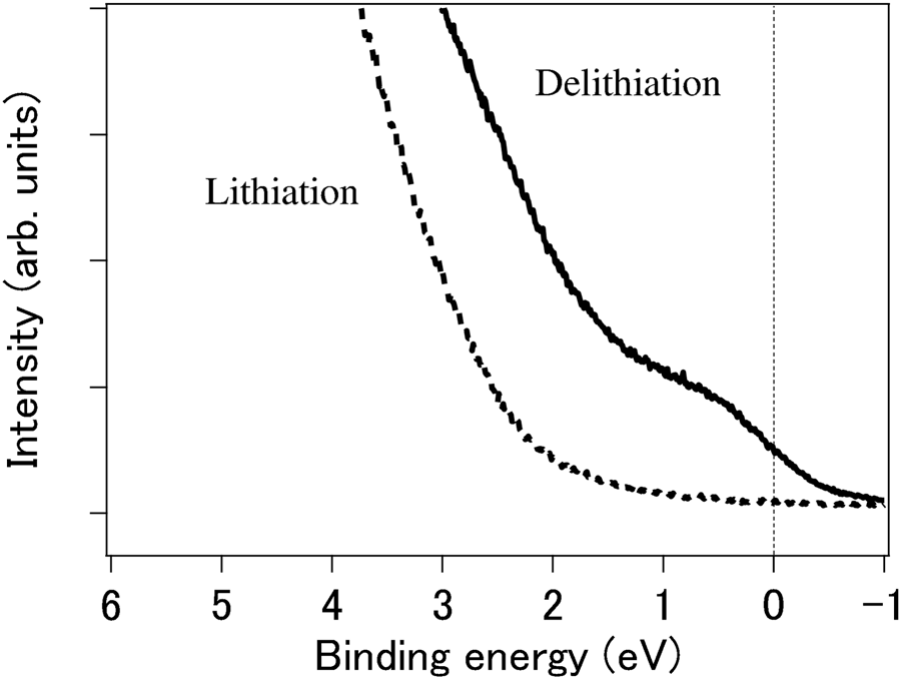
UPS spectra of the samples after delithiation (solid, *x* = 2) and lithiation (dashed, *x* = 10) at room temperature. The vertical line shows the Fermi level.

Next, we performed UPS to investigate the electronic states around the E_*F*_ (Fig. 4). No states near the E_*F*_ were observed for the lithiated sample (Li_10_FeS_5_); in contrast, finite states at the E_*F*_ were visible for the delithiated sample (Li_2_FeS_5_). The drastic change in the electronic states suggests switching between the insulating state and the conductive state by Li ions.

### Local structural models

**Figure 5:**
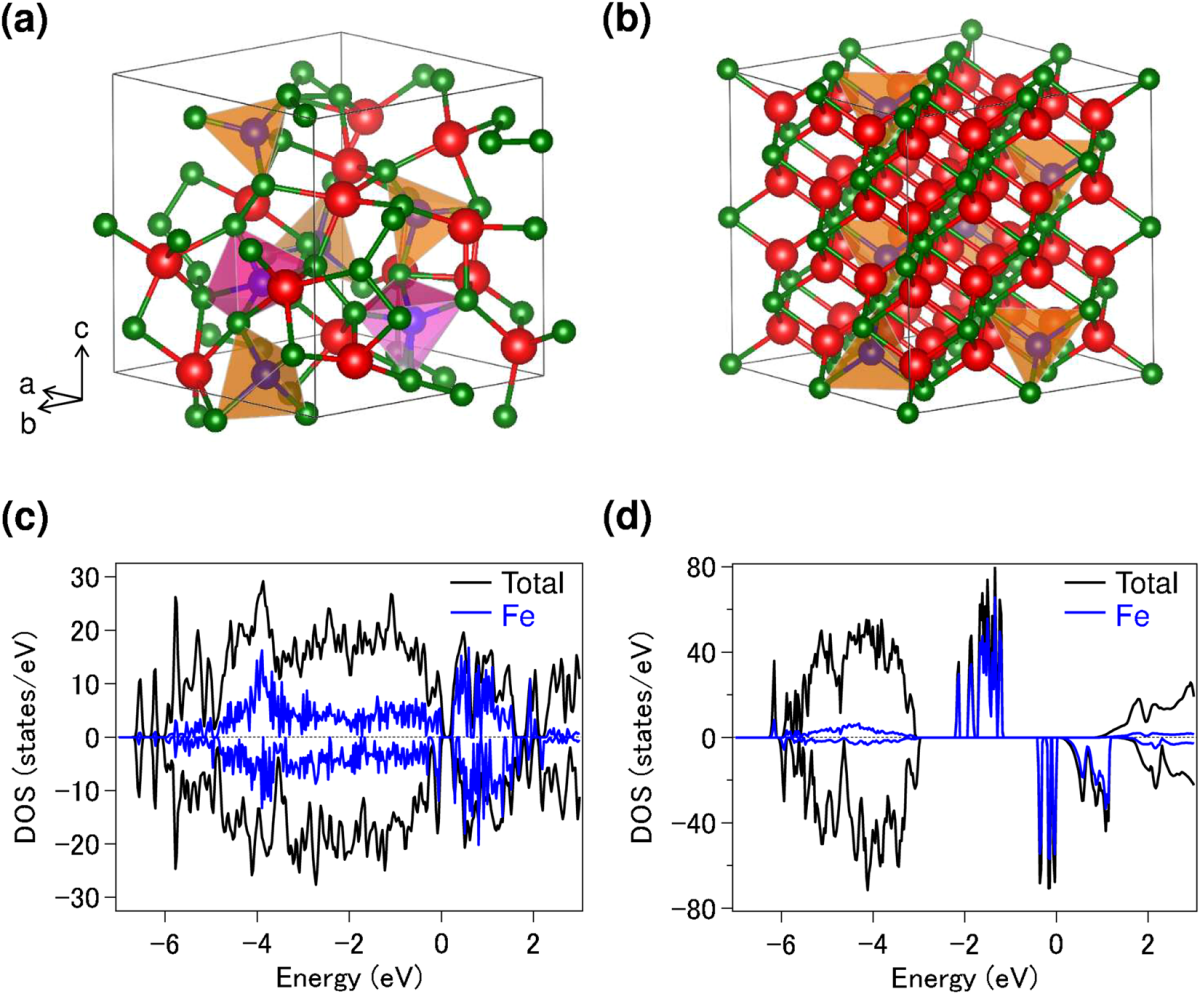
Models of local structures for (**a**) Li_2.2_Fe_0.94_S_5_ and (**b**) Li_9.1_Fe_0.94_S_5_. Fe–S clusters are shown by FeS_5_ trigonal bipyramids (purple) and FeS_4_ tetrahedra (brown). Lithium, iron and sulfur atoms are represented by red, blue and green spheres, respectively. Calculated density of states (DOS) for (**c**) Li_2.2_Fe_0.94_S_5_ and (**d**) Li_9.1_Fe_0.94_S_5_. Positive and negative values of DOS correspond to spin-up and -down, respectively.

Finally, the local structural models that we consider by DFT calculations are shown in Fig. 5a,b. Pristine Li_8_FeS_5_ crystallizes in the Fm$\overline{3}$m space group, in which the Fe ions partially occupy the Li sites in Li_2_S^9^. LiS_4_ and FeS_4_ tetrahedra are included in the lattice and they are edge-shared. Delithiation (lithiation) induces the following two unique local structural changes: (i) formation of FeS_5_ trigonal bipyramids (FeS_4_ tetrahedra) and (ii) formation (dissociation) of S–S bonds. When Li is extracted from the pristine material, partial Li–S bond breaking is facilitated and the discrete S–S bonds are formed. In addition, FeS_5_ trigonal bipyramids and FeS_4_ tetrahedra coexist in the lattice. Since Fe ions are generally coordinated either teterahedrally or ocahedrally, the local coordination geometry in Li_2.2_Fe_0.94_S_5_ is counterintuitive. The existence of FeS_5_ trigonal bipyramids is hard to envisage experimentally. Specially, to the best of our knowledge, this coordination in inorganic compounds is the rare example. The displacement of the central Fe in FeS_4_ tetrahedra is a key to explain the formation of FeS_5_ trigonal bipyramids because the 4S tetra-framework remains. In overall, the lack of long-range periodicity (amorphous structure) in the predicted structure (Fig. 5a) is consistent with the previous experimental report^8^. On the contrary, for Li_9.1_Fe_0.94_S_5_, each element is arranged more periodically than in Li_2.2_Fe_0.94_S_5_. As shown in Fig. 5b, the framework of Li_2_S is predicted to be stable in the lattice, although Li is partially replaced by Fe, resulting in the formation of FeS_4_ tetrahedra and the dissociation of isolated S–S bonds.

DFT calculations also predict that the spin-up and -down Fe states are almost symmetrical for Li_2.2_Fe_0.94_S_5_, indicating a non-magnetic nature (Fig. 5c). However, for Li_9.1_Fe_0.94_S_5_, there is a band gap and the overall DOS feature becomes partially asymmetrical (Fig. 5d). Such imbalance in the up and down spins gives rise to ferromagnetism. The valence band near the *E*_F_ is dominated by the Fe component. Spin polarization analyses suggest that the Fe^+^ ions are in the high-spin state (S = 3/2), which would be the origin of FM behavior. In addition, Bader charge analyses show that the average valences of Li, Fe and S ions are 0.77, 0.51 and −1.5, respectively. These results are reasonable each other. Although the prediction of the low valence state of Fe ions looks surprising, a low-valence state has also been predicted for Li_*x*_TiS_4_ (e.g., Ti^0.269+^; *x* = 4)^14^.

## Conclusion

In conclusion, Li ions drive a drastic phase transformation in the Li_*x*_FeS_5_ system. Delithiation and lithiation accompanied by up to eight Li ions transform pristine Li_8_FeS_5_ into a Pauli paramagnetic conductor (amorphous Li_2_FeS_5_) and a superparamagnetic insulator (low-crystalline Li_10_FeS_5_), respectively. The findings boost our still-limited understanding of the ionic perturbations of magnetism and conduction. By carefully selecting fragile/reconfigure materials, the ionic perturbations extend far beyond other ones to realize new phases and emergent properties.

## Methods

The primary electrochemical studies that carried out on Li_8_FeS_5_ have been reported elsewhere^8^. The bulk magnetization measurements were performed as described previously to avoid air exposure^15^. The local electronic resistance was measured by SSRM (AFM5000II, Hitachi High-Tech Science). The technique enables simultaneous local topographic and electronic resistance mapping on the surface of samples in real space. To confirm the repeatability, element distribution and absence of magnetic impurities, element mapping combined with SSRM (Park NX10, Park Systems) was performed using an Auger electron spectrometer (JAMP-9510F, JEOL) at room temperature. To investigate the electronic states near the *E*_F_ in detail, HeI (21.22 eV) UPS measurements were performed at room temperature (PHI5000 VersaProbe I, ULVAC-PHI). To detect probe – sample interactions and analyze the magnetic properties of the NPs after lithiation, we conducted MFM measurements (AFM5300E, Hitachi High-Tech Science) under vacuum conditions (6.6 × 10^−6^ torr) at 150 K. First-principles calculations were performed using the projector augmented-wave (PAW) method^16,17^. The exchange-correlation functional was used within the generalized gradient approximation (GGA-PBE)^18^. The selected energy cutoff was 340 eV, and Brillouin zone integration was performed on a 2 × 2 × 2 *k*-point mesh. The structural parameters were fully optimized until the atomic Hellmann-Feynman forces were less than 0.05 eV Å^−1^ and all stress components were less than 0.01 eV Å^−3^. To model the Li_2.2_Fe_0.94_S_5_ (Li_9.1_Fe_0.94_S_5_) material, a 2 × 2 × 2 supercell containing 52 (96) atoms was applied. The structure models were visualized using the VESTA program^19^.

## Supplementary information

Supplementary Information
