# Supplementary material for: Spin polarization in the phase diagram of a Li–Fe–S system
Source: Sci Rep. 2019 Dec 27;9:19947. doi: 10.1038/s41598-019-56244-x (PMC6934561; doi:10.1038/s41598-019-56244-x)
Supplement: Supplementary file 1 — Supplementary Information [file 41598_2019_56244_MOESM1_ESM.pdf]

SUPPLEMENNTARY MATERIALS

Spin polarization in the phase diagram of a Li–Fe–S system

Tsuyoshi Takami[1,*], Tomonari Takeuchi[2] and Toshiharu Fukunaga[1]

[1]Office of Society-Academia Collaboration for Innovation, Center for Advanced Science & Innovation, Kyoto University, Gokasho, Uji, Kyoto 611-0011, Japan
[2]Research Institute of Electrochemical Energy (RIECEN), National Institute of Advanced Industrial Science and Technology (AIST), 1-8-31 Midorigaoka, Ikeda, Osaka 563-8577, Japan

[*]Corresponding author:
Email: t-takami@saci.kyoto-u.ac.jp (T.T.)

1. Methods

Local electronic resistance was measured by SSRM (AFM5000II, Hitachi High-Tech Science). The technique enables simultaneous local topographic and electronic resistance mapping on the surface of samples in real space. The measured resistance is the sum of the local spreading resistance, the barrier resistance between the tip and the sample, the bulk resistance of the sample and the resistance of the tip. We employed a bias voltage of 10 V on a 10-mm diameter pellet prepared by cold pressing. The current through the tip/sample contact was monitored. The measurements were performed under vacuum conditions (7.5×10^{-7} torr) at room temperature. To confirm the repeatability, element distribution and absence of magnetic impurities, element mapping combined with SSRM (Park NX10, Park Systems) was performed at room temperature using an Auger electron spectrometer (AES) (JAMP-9510F, JEOL). To investigate the electronic states near the Fermi level in detail, HeI (21.22 eV) UPS measurements were performed at room temperature (PHI5000 VersaProbe I, ULVAC-PHI). To detect probe–sample interactions and analyze the magnetic nature of the nanoparticles (NPs) after lithiation, we conducted MFM measurements (AFM5300E, Hitachi High-Tech Science) under vacuum conditions (6.6×10^{-6} torr) at 150 K. The MFM experiments were carried out using the commercial piezoresistive cantilever (SI-MF20; spring constant k = 17 N/m, resonant frequency f = 136 kHz) as a force sensor. The phase shift $\Delta\Phi$ is given by a simple form

$\Delta\Phi \approx -(Q/k)(\partial F_z/\partial z)$,

where Q, k and F_z are the quality factor, the spring constant of the cantilever and the vertical component of the tip-sample interaction force, respectively. The MFM tip consists of an 80-nm layer of Co/Pt/Cr alloy, which is attached to the end of the cantilever. All scans were operated in a tapping mode. A linear mode may be suitable for imaging the SPM NPs, but the tip end may be destroyed due to direct contact of the tip with the sample surface because we performed the MFM measurements in pellet form. We applied an in-plane external magnetic field of 5 kOe.

2. Magnetization Measurements

Magnetization analysis above 250 K yielded a Curie temperature of 90 K and an unusually high spin quantum number (S = 16) (Fig. S1). After the second plateau at approximately 1.4 V in the lithiation process, the 2 K magnetization was prone to saturation at higher magnetic fields, but it did not saturate completely even at 70 kOe. A magnetic hysteresis with a residual magnetization was observed at 2 K, but it disappeared at 150 K (Fig. S2).

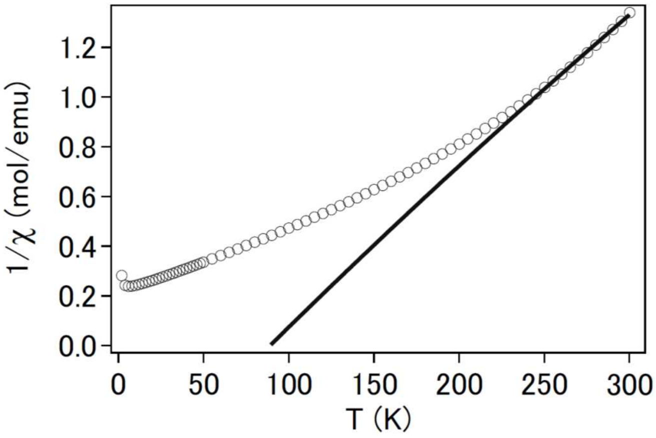

Figure S1. Inverse susceptibility plot for the sample after lithiation (x = 10 in Li_xFeS_5). The solid line is the fitted result with use of the Curie–Weiss law.

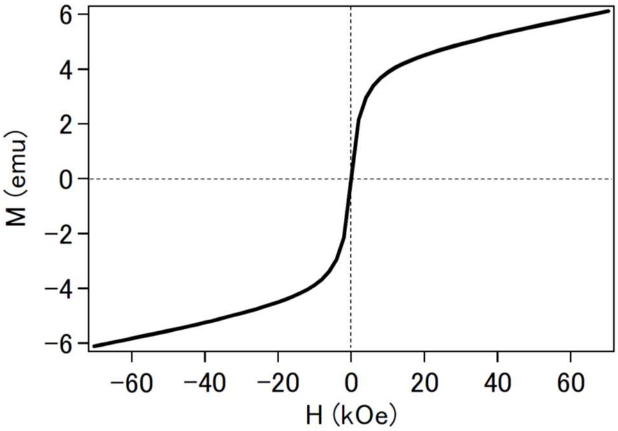

Figure S2. Magnetization as a function of magnetic field at 150 K.

3. SSRM Measurements

The results of element mapping combined with SSRM are shown in Fig. S3. The local resistance exhibited spatial variability. The low resistive region after delithiation is colored by black, while that after lithiation by brown. Therefore, it can be confirmed again that the resistance after delithiation is smaller than that after lithiation. The higher conductive regions correspond to the distributions of S and Fe signals. Fe signals were indistinct owing to a low excitation energy for AES mapping.

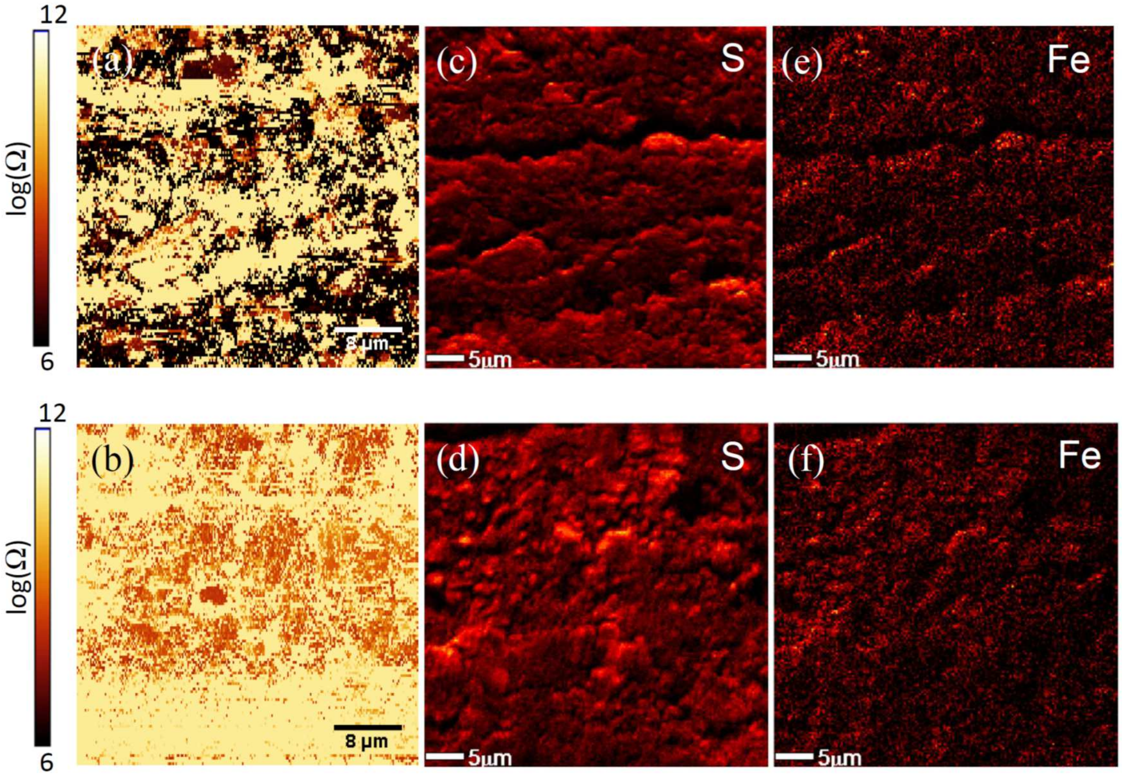

Figure S3. SSRM images of the samples after (a) delithiation and (b) lithiation. Scale bar: 8 µm. AES map of the same area after (c,e) delithiation and (d,f) lithiation. Elemental distributions of S and Fe are displayed. Scale bar: 5 µm.
